# Expression of Genes Related to Lipid Handling and the Obesity Paradox in Melanoma: Database Analysis

**DOI:** 10.2196/16974

**Published:** 2020-05-19

**Authors:** Claudia Giampietri, Luana Tomaipitinca, Francesca Scatozza, Antonio Facchiano

**Affiliations:** 1 Department of Anatomy, Histology, Forensic Medicine and Orthopedics Sapienza University of Rome Rome Italy; 2 Istituto Dermopatico dell’Immacolata - Istituto di Ricovero e Cura a Carattere Scientifico, IDI-IRCCS Rome Italy

**Keywords:** gene expression, obesity paradox, melanoma, colorectal cancer, CD36, FABPs, transcriptomic analysis, public databases

## Abstract

**Background:**

Publicly available genomic and transcriptomic data in searchable databases allow researchers to investigate specific medical issues in thousands of patients. Many studies have highlighted the role lipids play in cancer initiation and progression and reported nutritional interventions aimed at improving prognosis and survival. Therefore, there is an increasing interest in the role that fat intake may play in cancer. It is known that there is a relationship between BMI and survival in patients with cancer, and that there is an association between a high-fat diet and increased cancer risk. In some cancers, such as colorectal cancer, obesity and high fat intake are known to increase the risk of cancer initiation and progression. On the contrary, in patients undergoing treatment for melanoma, a higher BMI unexpectedly acts as a protective factor rather than a risk factor; this phenomenon is known as the obesity paradox.

**Objective:**

We aimed to identify the molecular mechanism underlying the obesity paradox, with the expectation that this could indicate new effective strategies to reduce risk factors and improve protective approaches.

**Methods:**

In order to determine the genes potentially involved in this process, we investigated the expression values of lipid-related genes in patients with melanoma or colorectal cancer. We used available data from 2990 patients from 3 public databases (IST [In Silico Transcriptomics] Online, GEO [Gene Expression Omnibus], and Oncomine) in an analysis that involved 3 consecutive validation steps. Of this group, data from 1410 individuals were analyzed in the IST Online database (208 patients with melanoma and 147 healthy controls, as well as 991 patients with colorectal cancer and 64 healthy controls). In addition, 45 melanoma, 18 nevi, and 7 healthy skin biopsies were analyzed in another database, GEO, to validate the IST Online data. Finally, using the Oncomine database, 318 patients with melanoma (312 controls) and 435 patients with colorectal cancer (445 controls) were analyzed.

**Results:**

In the first and second database investigated (IST Online and GEO, respectively), patients with melanoma consistently showed significantly (*P*<.001) lower expression levels of 4 genes compared to healthy controls: *CD36*, *MARCO*, *FABP4*, and *FABP7*. This strong reduction was not observed in patients with colorectal cancer. An additional analysis was carried out on a DNA-TCGA data set from the Oncomine database, further validating *CD36* and *FABP4*.

**Conclusions:**

The observed lower expression of genes such as *CD36* and *FABP4* in melanoma may reduce the cellular internalization of fat and therefore make patients with melanoma less sensitive to a high dietary fat intake, explaining in part the obesity paradox observed in patients with melanoma.

## Introduction

Genomic, transcriptomic, and proteomic data from several thousand patients and corresponding healthy controls are now publicly available on the internet, for many different pathologies, including different types of cancers. This allows researchers to investigate specific questions and medical hypotheses in silico, directly in the human context, without certain ethical concerns. We previously investigated expression data from several thousand patients, and identified novel potential markers useful for improving the diagnosis of melanoma and other solid cancers [1,2], as well as novel therapeutic approaches that were then validated in vitro by classical bench science [3,4]. In this study we aimed to determine the molecular mechanisms underlying the unexpected protective role of high fat intake in melanoma, given that obesity is a known risk factor in other cancers. The role fat plays in health maintenance as well as disease initiation and progression is being extensively investigated. There is particular interest in the protective role diet may have on cancer, since different cancer types are associated with being overweight or obese; furthermore, increased cancer mortality has been linked to dyslipidemia [5]. In patients with cancer, metabolic alterations impacting carbohydrate and lipid metabolism can activate phosphoinositide 3-kinase (PI3K) pathway–dependent oncogenic signaling, leading to an inflammatory state with increased expression of specific cytokines [6]. Omega-3 polyunsaturated fatty acids (PUFAs) have a beneficial effect by counteracting inflammation in cancer cells, which PUFAs easily diffuse into via the plasma membrane, by stimulating the production of anti-inflammatory metabolites [7]. PUFAs reduce plasma lipid levels and lipoproteins by modulating hepatic lipoprotein secretion [8] and likely by also mitigating dyslipidemia effects. How obesity and diet might impact melanoma onset and therapeutic efficacy has been discussed [9]. Although obesity and abnormal lipid levels in the blood represent established risk factors in other malignancies, they do not seem to impact cutaneous melanoma [10]. In fact, they are only slightly associated with an increased risk of cutaneous melanoma in men [11], although insulin resistance and dyslipidemia seem to promote the growth of uveal melanoma. Interestingly, obesity has been associated with a better prognosis and improved survival in patients undergoing treatment for metastatic melanoma. A higher BMI appears to be a protective factor in melanoma and this phenomenon has been named the obesity paradox [12]. In several other cancers, including colorectal cancer, being overweight and having a higher BMI are known risk factors, rather than protective conditions [13]. Fat metabolism might be differently controlled in different cancer cell types, thereby explaining why dyslipidemia may play divergent roles in different cancers. Scavenger receptors including macrophage receptor with collagenous structure (MARCO) and CD36 recognize and internalize lipoproteins, making them susceptible to degradation [14,15]. Furthermore, fatty acid–binding proteins (FABPs) play an important role in cancer progression and the intracellular transportation of long-chain fatty acids [16]. These molecules exert a pivotal role in regulating lipid metabolism.

The aim of this study was to investigate the expression of genes related to lipid-handling to analyze the molecular basis of the obesity paradox observed in melanoma.

## Methods

### Overview

The study was carried out in 3 steps: (1) a selection step was carried out on a public database, IST (In Silico Transcriptomics) Online, to identify genes of potential interest; (2) data collected in the initial selection step were validated in a first-round validation step with another database, GEO (Gene Expression Omnibus); and (3) data were further validated in a subsequent second-round validation step with a third database (Oncomine). Table 1 shows the databases used throughout the process and the types of patients investigated in each step.

First, in the selection step, the IST Online public database [17] was used to obtain gene expression data. It returns plots indicating the expression values of the given gene compared to the expression value of a second given reference gene. This can be carried out with several different cancer data sets and corresponding healthy controls. The analysis was performed with melanoma versus healthy skin and with colorectal cancer versus healthy control biopsies. In turn, we indicated our genes of interest (*CD36*, *MARCO*, *FABP1*, *FABP2*, *FABP3*, *FABP4*, *FABP6*, or *FABP7*) as the first gene and used a known housekeeping gene, beta-2 microglobulin (*B2M*), as the reference gene; it should be noted that the expression values of the first gene are independent from the reference gene and the values do not change if a different reference gene is chosen. We previously reported the methods used to study the expression of other molecules to identify relevant melanoma markers [2]. We analyzed data from 1410 individuals, including 208 patients with melanoma and 147 healthy controls, and 991 patients with colorectal cancer and 64 healthy controls. The first-round validation was carried out using the GEO public database [18]. The GDS1375 data set was used, which represents expression data from 45 melanoma biopsies, 18 nevi biopsies, and 7 heathy skin biopsies. The second-round validation was carried out on the DNA-TCGA data set in the Oncomine database [19]. In this case, 318 patients with melanoma were compared to 312 healthy controls, and 435 patients with colorectal cancer were compared to 445 healthy controls. The Human Protein Atlas public database was also interrogated [20].

**Table 1:** Schematic representation of the steps of this study.

Study phase and data set		Cancer type and subtype		Cancer samples, n	Control samples, n		Database
**Selection**						IST (In Silico Transcriptomics) Online	
	Melanoma and normal skin	Melanoma		208	147		
	Colorectal cancer and controls	Colorectal cancer		991	64		
**First validation round**						GEO (Gene Expression Omnibus)	
	Melanoma and normal skin (GDS1375 data set)	Melanoma		45	25		
**Second validation round**						Oncomine	
	DNA-TCGA	Melanoma		318	312		
	DNA-TCGA	**Colorectal cancer**					
			Colon adenocarcinoma	212	445		
			Rectal adenocarcinoma	90	445		
			Colon mucinous adenocarcinoma	37	445		
			Cecum adenocarcinoma	65	445		
			Rectal mucinous adenocarcinoma	7	445		
			Rectosigmoid adenocarcinoma	24	445		

### Statistical Analysis

Within the scatterplots obtained from the IST Online database analysis, the number of patients falling above or below the chosen threshold were counted and analyzed according to the Fisher exact test using GraphPad Prism 5 (GraphPad Software, Inc).

Other statistical analyses were carried out on the expression values obtained by querying the GEO database. Data was analyzed with analysis of variance and analysis for the linear trend from healthy to nevi to melanoma samples was carried out, both with GraphPad Prism 5. The threshold for statistical significance was set at *P*<.001.

## Results

### Overview

Gene expression of *CD36*, *MARCO*, and various *FABP* isoforms in 355 patients (208 patients with melanoma versus 147 healthy skin controls) was analyzed, according to the transcriptome expression data reported in the IST Online database. Table 2 shows the results and indicates the statistical significance of distribution above or below the given threshold, according to the Fisher exact test, for both melanoma and colorectal cancer data for all 8 genes investigated. The threshold value was chosen as the value best discriminating the largest population within the controls. The following threshold values were used: *CD36*: 1000; *MARCO*: 150; *FABP1*: 100 in melanoma and 1000 in colorectal cancer; *FABP2*: 100; *FABP3*: 250; *FABP4*: 2000; *FABP6*: 200; *FABP7*: 500. *FABP5* does not appear in the IST Online database.

Interestingly, the 4 genes that had a significant difference in melanoma were not significantly different in patients with colorectal cancer versus healthy controls, indicating that the difference observed in melanoma appears to be cancer-specific.

**Figure 1:**
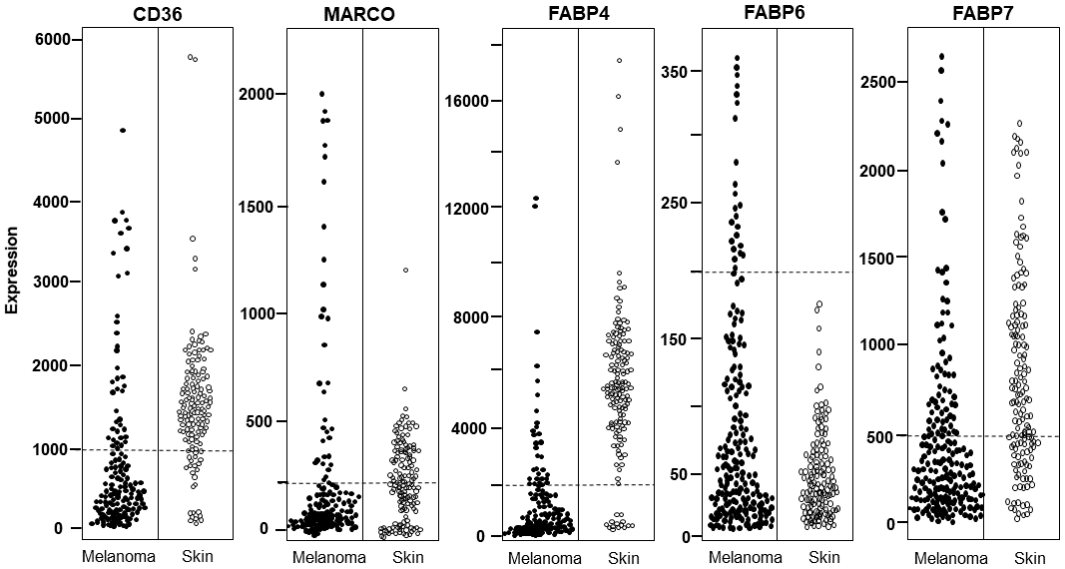
Expression of *CD36*, *MARCO*, *FABP4*, *FABP6*, and *FABP7* in melanoma and healthy skin samples from the IST (In Silico Transcriptomics) Online data set. Each dot indicates one individual and dashed lines indicate the threshold used to calculate the statistical significance of the distribution difference reported in Table 2. All reported genes show a significantly different distribution in melanoma versus controls according to the Fisher exact test (*P*<.001).

Figure 1 indicates the expression values of the 5 genes that had significant differences in melanoma versus healthy skin controls (*CD36*, *MARCO*, *FABP4*, *FABP6*, and *FABP7*). The significance of the distribution reported in Table 2 was computed by counting the number of individuals falling below or above the thresholds indicated by the dashed lines. The expression of *CD36*, *MARCO*, *FABP4*, *FABP6*, and *FABP7* in melanoma samples and healthy skin biopsies is visualized, according to data retrieved from the IST Online database.

These data indicate that melanoma samples show significantly lower expression of genes involved in fatty acid uptake (*CD36* and *MARCO*) and intracellular fatty acid binding (*FABP4* and *FABP7*) compared to healthy controls, and this phenomenon was not observed in a colorectal cancer data set.

**Table 2:** Expression in melanoma and colorectal cancer, according to the IST (In Silico Transcriptomics) Online database. Where *P* values are <.001, there was a statistically significant difference between the respective cancer values versus control values, evaluated as distribution above or below the given threshold, according to the Fisher exact test.

Gene	Regulation in melanoma versus controls	*P* value	Regulation in colorectal cancers versus controls	*P* value
*CD36*	Downregulation	<.001	No difference	.58
*MARCO*	Downregulation	<.001	No difference	.02
*FABP1*	No difference	.64	Downregulation	<.001
*FABP2*	No difference	.15	Downregulation	<.001
*FABP3*	No difference	.08	No difference	.07
*FABP4*	Downregulation	<.001	No difference	.60
*FABP6*	Upregulation	<.001	Upregulation	<.001
*FABP7*	Downregulation	<.001	No difference	>.99

### First Validation Round

**Figure 2:**
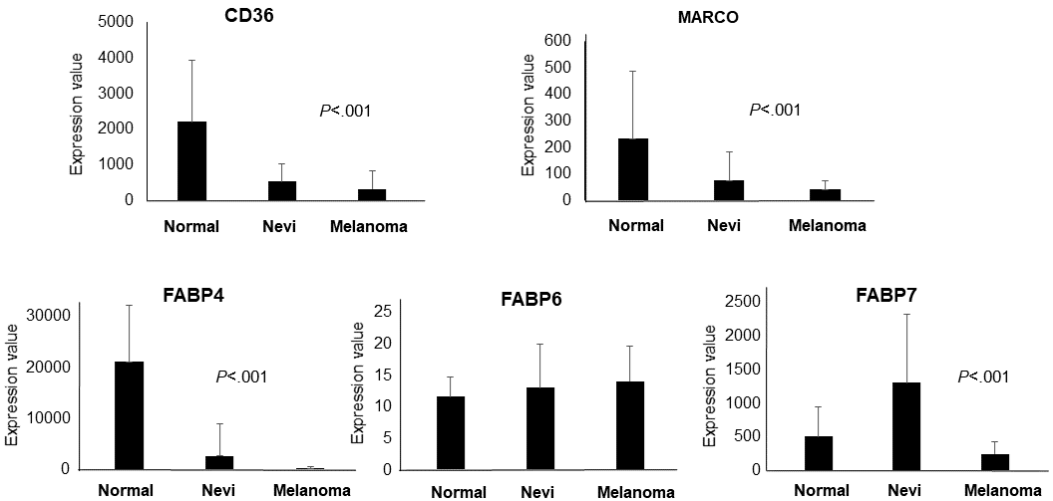
Expression of *CD36*, *MARCO*, *FABP4*, *FABP6*, and *FABP7* in melanoma, nevi, and healthy skin biopsies from the GDS1375 data set on the GEO (Gene Expression Omnibus) database. Changes observed in the GEO database were in the same direction as observed in the IST (In Silico Transcriptomics) Online database for all genes except *FABP6*. Statistical significance was calculated by analysis of variance.

Data collected from the IST Online database were then validated on a different database, GEO. Expression values in melanoma were obtained from the GDS1375 data set, as detailed in the Methods section. Figure 2 shows that the expression values of *CD36*, *MARCO*, *FABP4*, and *FABP7* are significantly decreased in melanoma samples (n=45) compared to nevi (n=18) and healthy skin (n=7) biopsies. A significant (*P*<.001) linear trend from healthy controls to nevi to melanoma biopsies was observed in *CD36*, *MARCO*, and *FABP4*. Therefore, the *CD36*, *MARCO*, *FABP4*, and *FABP7* data obtained from the IST Online database were validated on the GEO database. *FABP6*, which was increased in melanoma compared to control in the IST Online database (Table 2), showed a weak, nonsignificant increase in the GEO database (Figure 2).

### Second Validation Round

The 4 genes validated in GEO were further investigated in a third public database, Oncomine. Table 3 shows the statistical significance of the log2 copy number units change in the DNA-TCGA data set expression data from patients with melanoma and colorectal cancer compared to healthy controls, as analyzed in the Oncomine database. This analysis validated significant differences in *CD36* and *FABP4* expression in melanoma versus controls, and no significant difference in colorectal cancer.

**Table 3:** Gene expression in the DNA-TCGA data sets analyzed in the Oncomine database^a^.

Gene	*P* values for the melanoma DNA-TCGA data set	*P* values for the colorectal cancer DNA-TCGA data set
*CD36*	<.001	>.99
*MARCO*	.58	>.99
*FABP4*	<.001	>.99
*FABP7*	>.99	.04

^a^The statistical significance of the differential log2 copy number in patients with melanoma or colorectal cancer versus controls is reported. The significance threshold was set to *P*<.001.

A final investigation was then carried out using the Human Protein Atlas public database. Although the potential roles of the genes investigated in this study were not verified in melanoma, their roles have been confirmed in other cancers. Specifically, increased *CD36* gene expression levels indicate an unfavorable prognostic value in 354 patients with stomach cancer (*P*<.001), and increased *FABP7* gene expression levels indicate an unfavorable prognostic value in 877 patients with renal cancer (*P*<.001), yet indicate a favorable prognostic value in 1075 patients with breast cancer.

## Discussion

In this study we investigated the expression of different genes involved in lipid metabolism and found a significant difference in melanoma versus controls. This may explain part of the mechanism behind the obesity paradox observed in patients undergoing treatment for metastatic melanoma. The mechanisms underlying the association between dyslipidemia and melanoma remain controversial; this is due to the different metabolic controls within bulk melanoma cells and cancer stem cells or metastasis-initiating cells [21,22]. Metastasis-initiating cells display high *CD36* levels, which may indicate a crucial contribution of dietary lipids in the promotion of metastasis [23]. Furthermore, as we previously demonstrated, melanoma cancer stem cells show higher intracellular neutral lipids, higher lipogenesis activation, and lower autophagic flux [24]. This evidence indicates a complex molecular apparatus that allows melanoma cells to finely regulate fatty acid storage and mobilization depending on the metabolic environment and their differentiation level.

*FABP4* and *FABP2* have recently been reported to have a significant association with cancer progression in patients with colorectal cancer [16] and several studies demonstrate that obesity and high fat intake are risk factors in colorectal cancer [13,25-28]. On the other hand, several studies highlight the obesity paradox in melanoma, reporting significantly lower mortality in overweight patients undergoing treatment for melanoma [29], although a recent publication indicated that some caution is warranted [30]. Patients with melanoma or colorectal cancer appear to respond in opposite ways to high dietary fat intake or fat metabolism and may therefore be useful models to investigate how lipid-related gene expression may differentially regulate cancer initiation. For this reason, this study investigated lipid-related gene expression in patients with melanoma or colorectal cancer. Many genes have been identified as lipid modulators, although this field still remains poorly investigated. Genes controlling dyslipidemia in mice were recently reported [31], as well as other molecules that interfere with lipid storage [32,33], while a complete list of lipid-related genes in humans is currently lacking. In this work we investigated the expression of 8 genes (*FABPs* and other lipid-related genes) in melanoma and colorectal cancer biopsies, hypothesizing that differences in melanoma and colorectal cancer gene expression may partly explain the different role dietary fat plays in melanoma (ie, protective) and colorectal cancer (ie, detrimental). A significant reduction of the genes for scavenger receptors CD36 and MARCO (which are able to bind lipoproteins) and FABP4 and FABP7 translocases (which are able to bind and cell-internalize fatty acids) was found in melanoma biopsies compared to healthy controls, according to 2 independent databases, IST Online and GEO. We hypothesize that this reduced expression may lead to a reduced uptake of lipids and reduced cellular internalization. *CD36* and *FABP4* were also validated in a third database, Oncomine, using the DNA-TCGA data set. These genes showed no difference in control expression data compared to data from patients with colorectal cancer, for whom high fat dietary intake represents a negative prognostic factor. Therefore, we believe that the reduced gene expression observed in melanoma (571 patients and 484 controls, in 3 independent databases) might contribute to counteracting the detrimental effects of high fat intake.

More extensive analyses are ongoing in other cancers and on a larger list of relevant lipid-related genes; nevertheless, the results from this study may reveal some of the molecular mechanisms responsible for the obesity paradox observed in melanoma.

## Abbreviations

B2M: beta-2 microglobulin
FABP: fatty acid–binding protein
GEO: Gene Expression Omnibus
IST: In Silico Transcriptomics
MARCO: macrophage receptor with collagenous structure
PI3K: phosphoinositide 3-kinase
PUFA: polyunsaturated fatty acids
